# Characteristics of Color Produced by *Awa* Natural Indigo and Synthetic Indigo

**DOI:** 10.3390/ma2020661

**Published:** 2009-06-09

**Authors:** Miyoko Kawahito, Ryoko Yasukawa

**Affiliations:** 1 Tokushima Prefectural Industrial Technology Center/11-2 Nishibari, Saiga, Tokushima 770-8021, Japan; 2 Department of Chemistry and Materials Technology, Kyoto Institute of Technology/Matugasaki, Sakyo-ku, Kyoto 606-8585, Japan

**Keywords:** *Awa* natural indigo, synthetic indigo, brightness of color, running of color, color unevenness, color fading

## Abstract

Color of cloth dyed with *Awa* natural indigo is quantitatively compared with color of the cloth dyed with synthetic indigo. Results showed that: 1) color produced by *Awa* natural indigo is bluer and brighter than color produced by synthetic indigo; 2) a single Gaussian function fits the profile of the running of color produced by *Awa* natural indigo and the running of color produced by synthetic indigo prepared with sodium hydrosulfite approximates a linear sum of two Gaussian functions; 3) before and after washing, color is quantitatively more uneven when produced by *Awa* natural indigo than when produced by synthetic indigo; 4) the diffusion coefficient of *Awa* natural indigo is lower than that of synthetic indigo; 5) color superiority of *Awa* natural indigo relates to smaller diffusion coefficient, slower reduction, poorer penetration, and higher dye aggregation.

## 1. Introduction 

**Figure 1:**
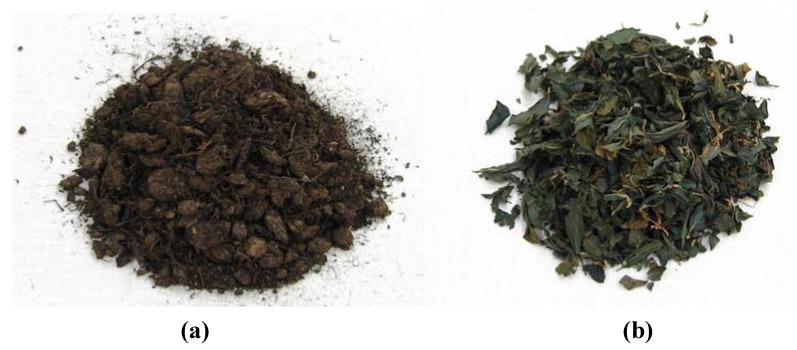
(a) *Awa* natural indigo. (b) Indigo dry leaves, which is materials of *Awa* natural indigo.

Traditional craft techniques are the basis of modern manufacturing in Japan, but nowadays those techniques are relatively rarely used because they are not so useful in modern industry. However, although synthetic indigo is the dye of choice for industry, particularly in the denim industry, the use of natural indigo and traditional techniques remains an important part of art and craft works [1]. Continuing a tradition that has lasted for centuries, natural indigo lovers maintain interest in that dyestuff’s unique color, variety of plants, dyes, and dyeing methods [2,3,4]. Blue color can be produced with the use of fresh indigo leaves, but using processed natural indigo dye is thought to produce deeper color and to preserve dye better [1]. One popular method for processing natural indigo dye features fresh indigo leaves soaked in water (called *doroai* or *chindenai* in Japan). Another method involves fermenting dry indigo leaves for about 90 days (called *sukumo* in Japan) [5]. Derived from *Persicaria tinctoria*, *Awa* natural indigo (Figure 1) is a form of *sukumo* that has been made in Tokushima (formerly known as *Awa*) region in Japan. Production has been about sixty tons per year for most years since 1985 [6]. 

Dyers who use *Awa* natural indigo emphasize its color rather than its history or rarity and feel that color of cloth dyed with *Awa* natural indigo differs from color of cloth dyed with synthetic indigo [7]. To try to explain scientifically how and why the colors produced by natural indigo and synthetic indigo can differ, *Awa* natural indigo was treated as a traditional natural indigo dye and synthetic indigo was treated as an industrial indigo dye reduced by zinc powder (a relatively weak reducing agent [8]) and sodium hydrosulfite (a relatively strong and common reducing agent for industrial indigo dyeing [9]). Color was compared using a spectrophotometer and a colorimeter and was correlated with dye diffusion and dye penetration. Comparison of *Awa* natural indigo and synthetic indigo was in terms of four key color characteristics: brightness of color [8,10], running of color [11], color unevenness [12], and color fading [12] and in terms of coloring materials in dye and dyed cloth, dye diffusion, dye aggregation, and dye penetration. 

**Figure 2:**
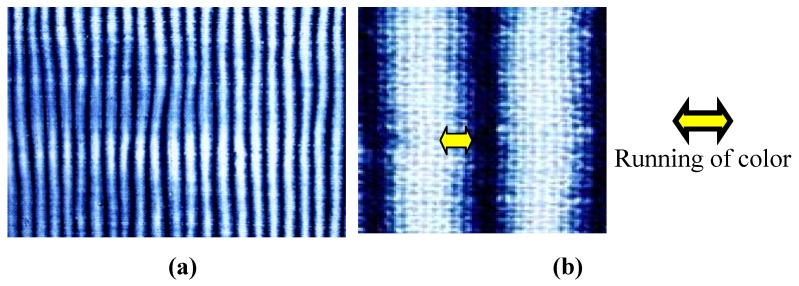
(a) Tie-dyed cotton cloth using a *bo-murakumo-shibori* method [11]. (b) Running of color [11].

In this paper, brightness of color includes nuances such as clarity, coolness, and color related to chroma. Running of color related to tie-dyeing with indigo. A *bo-murakumo-shibori* method [13] is used in this paper because its pattern is simpler and better for evaluation using scientific equipment. In the *bo-murakumo-shibori* method, cloth is stitched to form tube, a pipe is inserted in the central core of the cloth tube, and the cloth is compressed. After dyeing, the cloth that contacts the pipe produces a white area and the cloth that contacts the dye liquid produces a blue area. This method produces a pattern of alternating light (white) and shade (blue) (Figure 2a) and the color boundary between white and blue is known as running of color (Figure 2b) [14,15]. Running of color is an important component in the aesthetic beauty that is appreciated by some Japanese [16]. For example, one professional indigo dyer who used *Awa* natural indigo states that: “cloth dyed with synthetic dyes lacks something to move our heart, but running of color produced by natural indigo has purified beauty” [17].

In this paper, color unevenness that arises due to highly skilled dyeing technique is thought to be tasteful and positive. Color fading is seen as a positive element that appeals to the Japanese taste [9]. For example, one industrial artist believes that “through washing, cloth dyed with natural indigo changes beautifully with the cloth itself.” [18] 

## 2. Results and Discussion 

### 2.1. Color characteristics

Three dye baths were prepared and three cotton samples were dyed separately (Table 1).

**Table 1:** Samples and dye baths.

Sample	DyeBath	Indigo (Material)	Reducing agent
Sample No. 1	Dye Bath No. 1	*Awa* natural indigo	Fermentation
Sample No. 2	Dye Bath No. 2	Synthetic indigo	Zinc powder
Sample No. 3	Dye Bath No. 3	Synthetic indigo	Sodium Hydrosulfite

#### 2.1.1. Brightness of color

**Figure 3:**
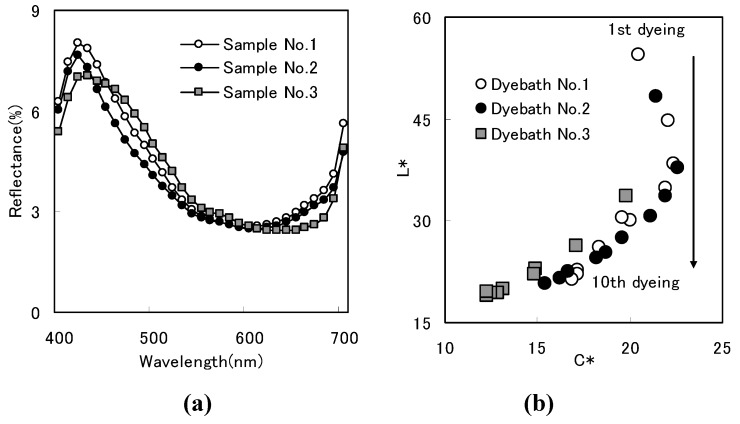
(a) Reflectance of dyed cloth [8]. (b) Color change due to repetitive dyeing [8].

To compare color brightness in samples with similar depth of shade, cloth reflectance was measured using a spectrophotometer. Maximum absorption wavelength was observed at 600 nm to 630 nm. Reflectance spectra confirmed that samples had similar depths of shade (Figure 3a). The spectrum distribution profile of Sample No.1 was sharper than in Sample No.2 and much sharper than in Sample No.3 (Figure 3a). Cloth with similar hue and similar lightness had spectrum distribution profile that was sharper with a higher chroma, resulting in greater difference between the peak and lowest level [19]. 

C^*^ (chroma) value and b^*^ value (direction of color, with b^*^ denoting yellow direction and -b^*^ denoting blue direction) were evaluated in a CIELAB color system using reflectance. Color characteristics were C^*^=16.90 and b^*^=-16.65 for Sample No. 1, C^*^=15.40, b^*^=- 15.10 for Sample No. 2, and C^*^=14.83 and b^*^=- 14.82 for Sample No.3. 

Cloth was dipped in an indigo dye bath and oxidized with air and repeating that process deepened color [5]. To examine color change due to repeated dyeing, L^*^ and C^*^ were plotted against the number of dyeing cycles. L^*^ denotes lightness. Chroma of Sample No. 1 was similar to the chroma of Sample No. 2 and higher than the chroma of Sample No. 3 (Figure 3b). 

#### 2.1.2. Running of color

**Figure 4:**
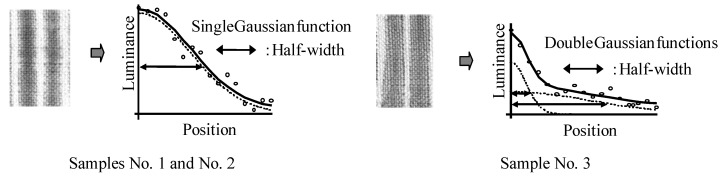
Luminance distribution curves fitted to Gaussian functions using reversed grayscale [11].

To compare running of color in hand woven cotton cloth, three tie-dyed samples were photographed, images were extracted and changed to reversed grayscale images, and luminance distribution curves were quantitatively analyzed in terms of Gaussian function. Similar luminance distribution profile appeared as a single Gaussian function for Sample No. 1 and Sample No. 2 and a linear sum of two Gaussian functions fitted reasonably well to the distribution profiles of Sample No. 3 (Figure 4). The linear sum of two Gaussian functions denoted that running of color is related to at least two independent mechanisms. This analysis indicated that running of color in cloth tie-dyed with *Awa* natural indigo seems to differ from running of color in cloth tie-dyed with synthetic indigo reduced by sodium hydrosulfite. 

Average luminance distribution was specified quantitatively by evaluating the corresponding half-width as a measure of the breadth of the luminance distribution. Half-width evaluated for Sample No. 1 (8.8 pixels) was narrower than the half-width evaluated for the Sample No. 2 (12.5 pixels) and much narrower than the half-width evaluated for the Sample No. 3 (14.3 pixels and 3.5 pixels). Distribution profiles represented by a linear sum of two Gaussian functions had two corresponding half-widths and longer running of color. *Awa* natural indigo had one half-width and shorter running of color.

#### 2.1.3. Color unevenness

**Figure 5:**
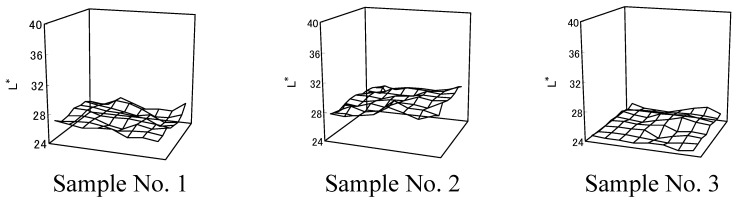
L* values in dyed cloth [12].

To analyze color unevenness quantitatively, L^*^ values of dyed cloth (448×384 points/40×40 mm^2^) were measured using a colorimeter. Because ambient light might affect measurements and color might appear to be mixed [20], the grid was divided into smaller cells and average L^*^ values in every cell (56×48 points/5×5 mm^2^) appeared on the z-axis (Figure 5). The x-axis and y-axis corresponded to the center position of a mesh in the dyed cloth. L^*^ data spread in Sample No.1 was more than L^*^ data spread in Sample No.3.

Variation coefficients (standard deviations divided by average values) denoted data spread from average value. Increased data spread indicated more color unevenness. Color unevenness was evaluated using variation coefficients (Sample No. 1=0.021; Sample No. 2=0.021; Sample No. 3=0.014). There was more color unevenness in cloth dyed with *Awa* natural indigo than in cloth dyed with synthetic indigo reduced by sodium hydrosulfite. 

#### 2.1.4. Color fading by washing

**Figure 6:**
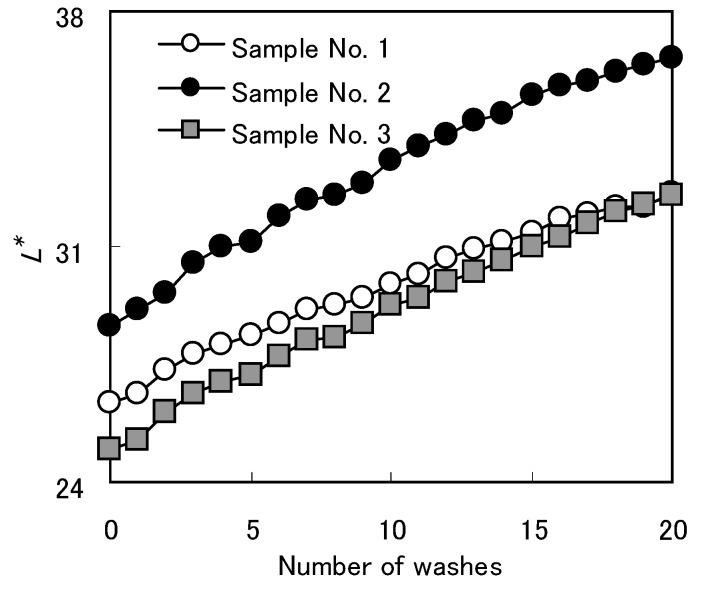
Number of washes and L* value of samples [12].

Color fading by washing in cloth dyed with *Awa* natural indigo was compared with color fading in cloth dyed with synthetic indigo. To examine color change caused by washing, averaged L^*^ values were plotted against the number of washes. The L^*^ values of cloth dyed with indigo, especially synthetic indigo, increased with repeated washing (Figure 6). 

**Figure 7:**
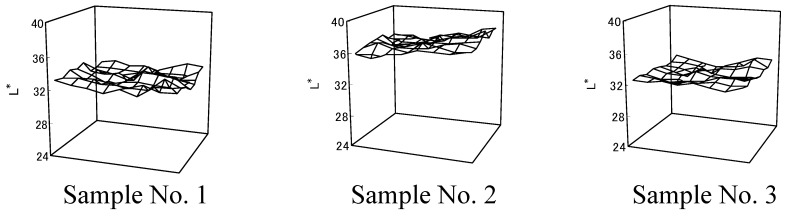
L^*^ values in dyed cloth after washing [12].

In every cell (56×48 points/5×5 mm^2^), distribution of average L^*^ value (Figure 7) and variation coefficients (Sample No. 1=0.018; Sample No. 2=0.017; Sample No. 3=0.015) showed more color unevenness in cloth dyed with *Awa* natural indigo than in cloth dyed with synthetic indigo. 

### 2.2. Coloring materials in dye and in dyed cloth

Generally speaking, natural indigo contains various impurities, including indirubin (an isomer of indigo), tannin and flavonoid [21,22]. Ushida *et al.* investigated *Awa* natural indigo using HPLC and found 3.2% indigo and 0.0018% indirubin [22]. The value of b^*^ significantly affected the value of C^*^ (see 2.1.1). Brightness of color depends on blue color materials rather than on red color materials. 

Coloring materials were extracted from two types (natural and synthetic) of indigo dye and three kinds of dyed cloth sample using acetonitrile, which does not readily dissolve indigo and dimethylformamide (DMF), which dissolve indigo well. Absorbance of the extracted solution was measured using an ultraviolet visible spectrophotometer (Nihonbunko Co. Ltd., Japan). 

Maximum absorption wavelengths of extracted dye solution using acetonitrile were 510 nm for *Awa* natural indigo and 548 nm for synthetic indigo [8]. Maximum absorption wavelengths of extracted solution using DMF were 610 nm for both dyes. Ratio of indirubin to indigo was < 0.06%. These results indicated that indigo in *Awa* natural indigo and synthetic indigo was identical in certain respects but coloring materials in the form of impurities differed in quality and/or quantity. The maximum absorption wavelength of the extracted solution from indigo using acetonitrile shifted to a shorter wavelength, confirming that coloring materials in impurities contained red coloring materials including indirubin. In samples exposed to acetonitrile, maximum absorption wavelengths of the extracted solution were: Sample No. 1 =596 nm, Samples No. 2 and 3 =594 nm. In samples exposed to DMF, maximum absorption wavelengths of the extracted solution were: Samples No. 1, 2, and 3 =610 nm [8]. 

In this paper, cotton cloth was used as the primary fiber material. However, because silk cloth is an important material for adding value to the *Awa* natural indigo dyed products, silk cloth samples were dyed using three different dye baths and examined regarding the effect on color by coloring materials other than indigo. Results showed that silk had more functional groups with high affinity to dye than other fibers [23], but no difference was seen in the maximum absorption wavelength in silk cloth dyed with indigo using two solvents (acetonitrile; Samples No. 1 and 3 =595 nm, Sample No. 2 =594 nm: DMF; Samples No. 1, 2, and 3 =610 nm) [10]. Silk could not be dyed with coloring materials other than indigo. 

Results indicate the coloring materials other than indigo had minor color effect on dyed cloth. There was little observable difference in maximum absorption wavelength for all cotton and silk samples. Coloring materials included as impurities may not affect cloth color during the dyeing process. 

### 2.3. Dye diffusion phenomenon

**Figure 8:**
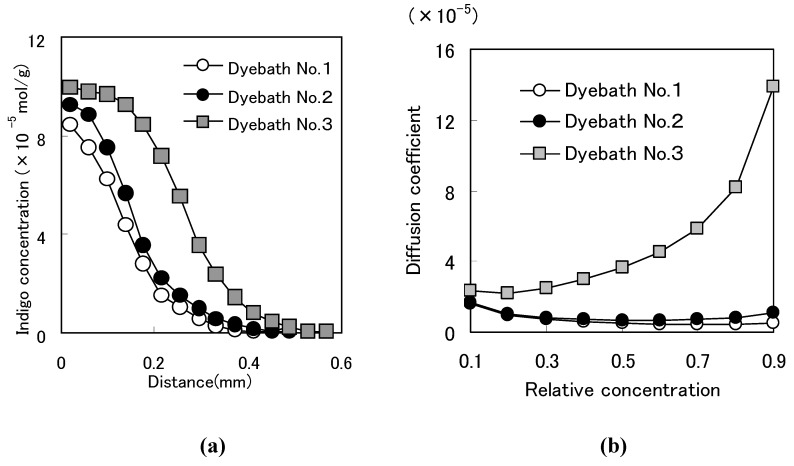
(a) Diffusion profiles of indigo [8]. (b) Diffusion coefficients and relative concentration.

Penetration of indigo into cellophane film was examined to investigate the dyeing process of *Awa* natural indigo and synthetic indigo [24]. Figure 8a shows diffusion profile of indigo based on indigo concentration in each layer of cellophane film and a penetration distance from the dye liquid. Indigo concentration in the film was calculated based on absorbance (Figure 8a). 

Synthetic indigo reduced by sodium hydrosulfite penetrated deeper than synthetic indigo reduced by zinc powder and much deeper than *Awa* natural indigo. Poor penetration of indigo related to week reduction as indigo in the Dye Bath was well oxidized by water in the cellophane film. Greater penetration of indigo related to strong reduction as indigo in the Dye Bath was not well oxidized by water. 

Diffusion coefficients and relative concentration showed, for example, Dye Bath No.1=4.942×10^-6^ cm^2^/min, Dye Bath No.2=6.797×10^-6^ cm^2^/min, Dye Bath No.3=3.631×10^-5^ cm^2^/min at relative concentration of 0.5 (Figure 8b). These results show that diffusion coefficients of synthetic indigo are higher than in *Awa* natural indigo, especially when sodium hydrosulfite is used. Figure 8b also indicates deep penetration of synthetic indigo. Synthetic indigo reduced by sodium hydrosulfite penetrated deeper than synthetic indigo reduced by zinc powder and much deeper than *Awa* natural indigo. 

### 2.4. Dye aggregation

Apparent molecular weight of indigo in dye baths was measured to evaluate the degree of aggregation using Ultrafilter 5000 and Ultrafilter 30000. Measurements of absorption spectrum of filtrates showed that Ultrafilter 30000 could not filter Dye Bath No.1 but Ultrafilter 5000 could filter Dye Baths No. 2 and No. 3. Apparent molecular weight of *Awa* natural indigo was over 30,000 and apparent molecular weight of synthetic indigo was less than 5,000.

### 2.5. Dye penetration

**Figure 9:**
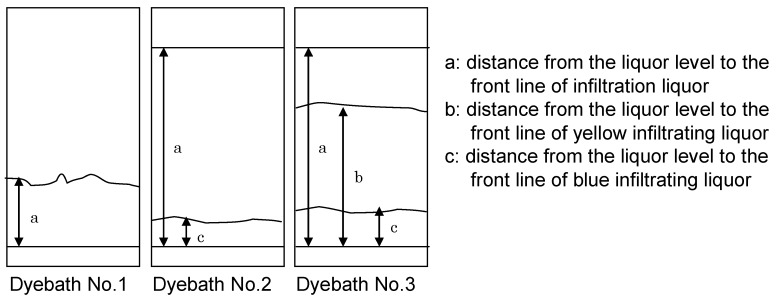
Dye penetration using filter paper [11].

To examine dye penetration, filter paper was used as a model for cotton cloth without fabric texture (Figure 9). The distance between the liquor level and the line of furthest liquor infiltration was measured to evaluate dye penetration in adsorbent (filter paper) and that distance was found to be shorter in Dye Bath No.1 than Dye Bath No.2 and Dye Bath No.3. Results confirmed the presence of larger aggregations of indigo in Dye Bath No.1 and showed poorer penetration in Dye Bath No. 1 than in Dye Baths No. 2 and No. 3. 

Yellow and blue filtrating liquor differed in Dye Bath No.2 and Dye Bath No. 3. With drying, the yellow area (reduced indigo) eventually turned blue. Aggregated indigo clusters (blue) infiltrated slowly, but stronger reduction by sodium hydrosulfite caused further reduction of indigo that penetrated deeper and was observed as yellow infiltrating liquor. Reducing agent determined depth of indigo penetration into fiber.

### 2.6. Color and dyeing process

Reduction in Dye Bath No.1 was slower than in Dye Bath No.2 and much slower than in No. 3. Aggregation in Dye Bath No.1 was much more than in Dye Baths No. 2 and No. 3. Dye penetration in Sample No.1 was poorer than in Sample No.2 and much poorer than in Sample No.3. Brightness of color, running of color, color unevenness, and color fading in cloth dyed with synthetic indigo reduced by zinc powder (weak reducing agent) were between the amount produced by *Awa* natural indigo reduced by fermentation and the amount produced by synthetic indigo reduced by sodium hydrosulfite. These results confirmed differences between *Awa* natural indigo and synthetic indigo in terms of the four key color characteristics. 

Color brightened as reflected light decreased and permeated light increased [25]. Slower reduction by traditional fermentation in *Awa* natural indigo increased dye aggregation. More indigo gathered on fiber, producing brighter color than in synthetic indigo. Slower reduction by traditional fermentation in *Awa* natural indigo produced higher dye aggregation, poorer dye penetration, and shorter running of color than the faster reduction in synthetic indigo. 

Slower reduction and higher dye aggregation caused absorption of oxidized dye on fiber surfaces and caused color unevenness. Higher aggregation of *Awa* natural indigo produced more color unevenness than synthetic indigo with lower aggregation. Due to poorer penetration, in samples with similar depth of shade, cloth dyed with *Awa* natural indigo contained less dye than cloth dyed with synthetic indigo. Color change in *Awa* natural indigo with poorer penetration was less than color change in synthetic indigo.

## 3. Experimental Section 

### 3.1. Materials

Commercial synthetic indigo reagent (Kantoukagaku Co,. Ltd.), *Awa* natural indigo made by indigo farmers in Tokushima, hand woven cotton cloth (Tanakanao-Senryoten Co., Ltd.), ultrafilter (Nihon Millipore Co., Ltd.), and filter paper (Type 2, Toyo Roshi Kaisha) were prepared. Standard cotton and silk clothes for dyeing (JIS L 0803 [1998] and hand woven cotton cloth were boiled in distilled water for 1 hour, and rinsed with distilled water. Cellophane film (Rengo Co., Ltd.) was boiled in distilled water for 1 hour, left in water for 16 hours, and rinsed with distilled water.

### 3.2. Dye Bath

Dye Bath No.1 contained *Awa* natural indigo reduced by fermentation. Reduction by fermentation follows a traditional method using a bacillus that is found in *Awa* natural indigo instead of using a chemical reducing agent. 1) *Awa* natural indigo (8.4 Kg), alkali water (35 L) based on wood ash, slaked lime (150 g), and *sake* (270 cc) are put in a container (75 L) and stirred. 2) A few days later when pH<10.5, alkali water (30 L) and slaked lime (90 g) are added and stirred. 3) A few days later when pH<10.5, slaked lime (60 g) is added and stirred. 4) Stirring continues daily. This reduction processes is sometimes delayed or hastened. A few days after the processes completed, dyeing begins. 

Dye Bath No.2 and Dye Bath No.3 contained two types of synthetic indigo reduced by adding 2 g/L zinc powder and 1.25 g/L calcium oxide and reduced by adding 7 g/L sodium hydrosulfite and 2.5 g/L sodium hydroxide, respectively. Three dye baths were prepared to provide similar indigo concentration. *Awa* natural indigo concentration was calculated from the absorbance extinction measured by an ultraviolet visible spectrophotometer (Nihonbunko Co. Ltd., Japan) according to a calibration curve representing the relationship between indigo concentration and absorbance extinction of dissolved indigo reagent in a diluted DMF solution (DMF:H_2_O=9:1). 

### 3.3. Reflectance

Cloth reflectance was measured at the wavelengths between 400nm and 700nm with 10 nm intervals by a spectrophotometer CM-3700d (Minolta Co. Ltd., Japan), and analyzed by a CIELAB color system (JIS Z 8729 [1994]). A mean and standard deviation of ten measurements were calculated (standard deviations were: δL^*^=0.0053, δa^*^=0.0114, and δb^*^=0.0057). *R* denotes reflectance.

### 3.4. Diffusion phenomenon using cylindrical cellophane film rolled method

To prepare a film roll, cellophane film (0.039mm thickness when wet) was rolled on to a glass tube and paired with a glass tube that was not covered with film. The pair of tubes was fastened together with rubber bands and dyed. The film roll was dipped for 5 hours in each of the three dye baths, aired, rinsed and dried. 

Concentration of indigo in each layer of cellophane film was evaluated from the measured absorbance extinction according to the calibration curve. Diffusion coefficients were calculated according to the method proposed by Sekido [24] , Equation (1).

(1) $$dC/dt=Dd^{2}C/dx^{2}\;(C=indigo\;concentration,\;x=distance,\;D=diffusion\;coefficient)$$

### 3.5. Evaluation of running color

Tie-dyed cloth samples were photographed under fluorescent room light using a digital camera Olympus Camedia C-3030 Zoom (Olympus Optical Co., Ltd., Japan). Photographs of the running of color were taken at a distance of 30 cm using F4.5 iris diaphragm and a shutter speed of 1/100 sec. 100×150 pixel (1 mm=5 pixels) images were extracted at ten arbitrary positions of running of color using software Image-Pro Plus (Media Cybernetics, Inc., USA). 

Luminance was determined after conversion to grayscale image that was reversed so that the most deeply dyed part appeared as the brightest in the image data. Average one-dimensional luminance distribution represented luminous change from a blue stripe to a white stripe. Luminance distribution curves were quantitatively analyzed in terms of the Gaussian function using software Igor Pro (Wave Metrics, Inc., USA). Luminance distribution curves were represented by the curve of composed Gaussian functions (2). A half-width was calculated from the Gaussian Equation (2).

(2) $$F(x)=a\cdot exp\;\{-{\left[(x-b)/c\right]}^{2}\}+d\cdot exp\{-{\left[(x-b)/e\right]}^{2}\}+\cdot \cdot \cdot$$

### 3.6. Dye penetration

The lower end of filter paper (3cm) was dipped in three dye baths and infiltrated with liquor for 30 minutes. The distance between the liquor level and the front of infiltrating liquor was measured.

### 3.7. Apparent molecular weight

Dye baths were filtered through an Ultrafilter 5000 and an Ultrafilter 30000. Dye baths and filtrates were treated by diluted DMF aqueous solution (DMF:H2O=9:1). Absorption spectrum was measured in the range from 500 nm to 700 nm by UV-visible recording spectrophotometer UV-160A (Shimadzu Co., Ltd., Japan).

### 3.8. Method of washing

Samples were washed according to the test method for color fastness (JIS L 0845[1975]), dipped for 10 minutes in their respective aqueous baths with 50:1 liquor ratio (97±2 degrees Celsius) and dried in a drier (70-75 degrees Celsius). Dried samples were pressed (160 degrees Celsius) for 30 seconds using a flat bed press Type FB-2 (Yasuda Seisakusho Ltd.). 

## 4. Conclusions 

This paper confirms that the color of *Awa* natural indigo is somewhat brighter and bluer than that of synthetic indigo reduced by zinc powder (weak reducing agent) or sodium hydrosulfite (strong reducing agent). Experiments reported in this paper emphasize that running of color is a key characteristic of cloth dyed with *Awa* natural indigo and support the commonly held view that the color of cloth dyed with *Awa* natural indigo is brighter than the color of cloth dyed with synthetic indigo. The experiments also support the commonly held feeling that products dyed with *Awa* natural indigo have individuality which mass-produced industrial products lack. 

*Awa* natural indigo dye collects on the surface of fiber because of relatively poor penetration. Higher aggregation of the dye can produce uneven dyeing that is regarded negatively in industrial dyeing but positively in *Awa* natural indigo dyeing. Color unevenness depends on dye migration. Higher dye aggregation and less reduction produce lower dye migration and increase dye absorption on the surface of fiber. *Awa* natural indigo dye produces more aggregation than synthetic indigo dye produces. Experiments showed that preference for the color of cloth dyed with *Awa* natural indigo remains even after repeated washing and that color change depends on the amount of indigo dye in fiber. 

Results in this paper support the conclusion that reduction affects color in cloth dyed with indigo. The results show evidence of subtle color difference between *Awa* natural indigo and synthetic indigo and explain, at least partly, why the color of natural indigo may be considered superior to the color of synthetic indigo. Apparently, while Japanese industrial dyeing enjoys high evaluation worldwide, the industry still can not reach a level that can successfully represent sophisticated Japanese aesthetic concepts. 
